# Epidemiology and burden of adult chronic pancreatitis in South Australia: a 20-year data linkage study

**DOI:** 10.1136/bmjopen-2024-089297

**Published:** 2025-03-06

**Authors:** Tristan J Bampton, John W Chen, Alex Brown, Meghan I Barnett, P Toby Coates, Lyle John Palmer

**Affiliations:** 1 School of Public Health, The University of Adelaide, Adelaide, South Australia, Australia; 2 Department of Surgery, Flinders Medical Centre, Bedford Park, Australia; 3 Australian National University, Canberra, Australia; 4 School of Medicine, The University of Adelaide, Adelaide, South Australia, Australia; 5 Central Northern Adelaide Renal and Transplantation Service, Royal Adelaide Hospital, Adelaide, South Australia, Australia

**Keywords:** Pancreatic disease, Chronic Disease, EPIDEMIOLOGY, HealthCare Costs

## Abstract

**ABSTRACT:**

**Objectives:**

To investigate the epidemiology and burden of adult-onset chronic pancreatitis (CP) in South Australia.

**Design:**

Retrospective case-control study; data linkage.

**Setting:**

All public adult hospitals in SA.

**Participants:**

Administrative data linkage from South Australia-Northern Territory DataLink was used to ascertain an index cohort of all adults with an initial diagnosis of CP aged >19 years between June 2000 and June 2019. Age- and sex-matched controls were drawn from the general population of SA, adults with type 1 diabetes mellitus and adults with type 2 diabetes mellitus (defined by International Classification of Diseases 10th Revision coding).

**Main outcome measures:**

Hospital visits, days in hospital, emergency department visits, intensive care unit admissions, incidence, prevalence.

**Results:**

A total of 2503 incident index cases with CP were identified. The crude prevalence and incidence were estimated as 195.1 per 100 000 and 10.4 per 100 000 per annum, respectively. Cases of CP averaged more hospital visits for any reason (median 11, IQR 5 to 21.75) than the general population (median 1, IQR 0 to 4) and had a higher healthcare burden than controls with type 1 diabetes or type 2 diabetes (all p<0.001). Indigenous individuals were over-represented in the cohort (n=358; 14.8% vs 1.5% of the general population) and had higher healthcare utilisation than other patients with CP (p<0.001).

**Conclusions:**

CP is a significant burden on the SA healthcare system and was more prevalent and more burdensome in Indigenous adults. CP consumes a disproportionate level of public health services. Our findings support further research and preventive efforts, particularly in the Indigenous population.

**STRENGTHS AND LIMITATIONS OF THIS STUDY**

- Adult chronic pancreatitis (CP) has not been previously described epidemiologically in Australia.
- Our sample represents whole-of-population CP diagnoses in South Australian (SA) public hospitals, including in-patient and emergency department presentations over a 20-year period.
- Three age- (±1 year) and sex-matched general population controls were selected from all adult residents in SA registered on the Australian Electoral Roll.
- Up to three age- and sex-matched disease controls were drawn from (1) adults with type 1 diabetes mellitus and (2) adults with type 2 diabetes mellitus.
- Disease burden may have been overestimated as sampling frame was from hospital patients, and the most severe patients were more likely to have been managed in hospital.

## Introduction

 Chronic pancreatitis (CP) is a debilitating disease encompassing a spectrum of chronic inflammatory disorders of the pancreas.^1^ It is usually an acquired condition, often related to alcohol or cholelithiasis, with onset typically in the fifth to sixth decade of life.^2^,^4^ Between 1 and 4% of cases have a genetic aetiology with onset in childhood.^5^ Irrespective of aetiology, the natural history of CP typically follows a common pathological pathway. Recurrent episodes of ‘acute’ pancreatitis (AP) precipitate cycles of inflammation and resolution, causing irreversible damage through fibrosis and pancreatic parenchymal loss. A proportion of patients may present with CP without having previous episodes of AP.^6^

Diagnosis of CP is based on history, laboratory evidence of reduced pancreatic endocrine or exocrine function and radiological evidence of pancreatic fibrosis.^7^ Abdominal pain accounts for most CP-associated disease burden, both financially and regarding quality of life.^8^ Later manifestations include malabsorption with steatorrhoea due to impaired pancreatic exocrine function, and endocrine insufficiencies causing diabetes mellitus.^9^ The inflammation associated with CP is a significant risk factor for pancreatic cancer.^10^

Regardless of aetiology, the mainstay of CP management is supportive medical therapy. This involves analgesia, pancreatic enzyme replacement and insulin for those who develop diabetes.^11^ Up to 66% of patients require long-term opioid prescriptions,^12^ therefore risking opioid dependence and addiction, particularly for those with common comorbid addictive disorders such as alcoholism.^13^ Interventional therapies such as endoscopic stenting can achieve short-term relief; however, definitive surgery (partial or total pancreatectomy) may be required for effective long-term pain control.^14^ While these procedures provide durable relief from pain, they are associated with significant morbidity and mortality including ‘type 3c’ or pancreatogenic diabetes.^15 16^

A better understanding of the epidemiology and the relative burden of CP is important to inform policy and health services with regard to the future of CP management. Therefore, the specific aims of this study were to (1) describe the epidemiology of adult-onset CP in South Australia (SA) and (2) estimate the burden of adult-onset CP on the health system and society. These aims were achieved through the extraction of linked administrative data on the SA population covering the 20-year period from 2000 to 2019.

## Methods

The creation of the study population and associated linked administrative data was undertaken using the South Australia-Northern Territory (SA-NT) DataLink.^17^ SA-NT DataLink enables research access to administrative data generated by the State and Commonwealth Governments on the SA population. This organisation is part of an Australian-wide data linkage network.^18^

The sampling frame included all urban and rural SA public hospitals. The index case cohort consisted of all individuals with an initial diagnosis of CP at age >19 years from 1 June 2000 until 30 June 2019. The inclusion age was set to >19 years to match the Australian Census definition of ‘adult’.^19^ The inclusion criteria for a case was an International Classification of Diseases 10th Revision^20^ (15) (ICD-10) diagnosis of either ‘alcohol-induced CP’ (K86.0) or ‘other CP’ (K86.1). The index cases were identified through recorded diagnoses on inpatient separation summaries and emergency department (ED) visits, available as digital records from all SA public hospitals since 1 June 2000. Although the M-ANNHEIM classification system for CP^21^ is not formally used in Australia, SA public hospitals routinely use the radiological changes described in the M-ANNHEIM criteria to assist with diagnosis.

Three age- (±1 year) and sex-matched general population controls were selected from all adult residents in SA registered on the Australian Electoral Roll (registration to vote is compulsory in Australia). Up to three age- and sex-matched controls were drawn from: (1) adults with type 1 diabetes mellitus and (2) adults with type 2 diabetes mellitus. If the index case was recorded as being of Aboriginal or Torres Strait Islander descent, controls were also matched on ethnicity. There is often an overlap with diagnosing AP and CP, and therefore all diagnoses of AP (ICD-10 root code K85) were identified as a separate control group.

All available data on each index case and matched controls were requested from the custodians of the following linked datasets:

- SA inpatient separation summaries
- SA ED visit data collection
- Ambulance use
- SA births, deaths and marriages registry
- Socio-Economic Indexes for Areas.

Linked administrative data on the cases and controls were used to quantify healthcare burden. Key health summary indicators included: total hospital admissions; total days in hospital; total intensive care unit (ICU) admissions; total hours spent in ICU; median total hours spent in ICU over all admissions (with at least one admission); ED visits; total ‘procedures’ in hospital and any in-hospital interaction, including allied health interventions, anaesthetisation, blood transfusion, endoscopy, etc. Socioeconomic status (SES) was measured using the Index of Relative Socio-economic Advantage and Disadvantage (IRSAD). The IRSAD is produced by the Australian Bureau of Statistics and ranks areas in Australia according to relative socioeconomic advantage and disadvantage based on information from the 2016 Australian Census.^22^

The denominator for prevalence estimates was derived from the most recently available SA Census data (2016) and the numerator from the total number of index cases in the dataset. Subgroup analysis was performed to investigate the epidemiology and costs of CP in the Aboriginal and Torres Strait Islander population.

### Patient and public involvement

This research arose after clinical review of patients with CP in SA and interest in hereditary pancreatitis, a rare but important cause of pancreatitis in our community. Specific interviews with families of individuals with hereditary pancreatitis led to the question of raising broader awareness of pancreatitis in our state. Patient’s families were involved around the question of the broader impact of pancreatitis on healthcare, specifically around attendance at EDs in the public health system. Patients were not directly involved in recruitment as the current study used de-identified linked administrative data to identify cases. As participants are de-identified, the results of the study will be disseminated via scientific publication and local media release. As our previous work identified First Nations people as over-represented with pancreatitis, the project was discussed with the Aboriginal Health Council of South Australia, and specific Indigenous healthcare experts were consulted during the development of the project led by Professor Alex Brown. As a formal patient support group for First Nations people with pancreatitis does not currently exist, we discussed the project and needs of consumers with our local First Nations Kidney Reference Group (Aboriginal Kidney Care Together Improving Outcomes Now).

### Statistical analysis

Statistical analysis was performed using the R v4.0.0^23^ and Minitab v18^24^ statistical software packages. The study population was reported according to the RECORD guidelines.^25^ Summary statistics for continuous variables are presented as median and IQR (IQ1–IQ3). Summary statistics for categorical variables are presented as frequencies and percentages (n, %). The continuous outcome variables were: the number of ED visits, hospital procedures, hospital in-patient days, hours spent in ICU and IRSAD centile. The outcome ICU admission was coded as a binary variable (0=no admission, 1=≥1 admission). The principal explanatory variables were categorical case-control status. Bivariate statistical analysis investigating the association of continuous variables with binary variables was performed using Mann-Whitney U tests to account for the skewed distribution of the continuous variables. Bivariate statistical analysis of pairs of categorical variables was performed using contingency tables and χ^2^ tests. Subgroup analyses were performed in the (a) alcohol-induced patients with CP compared with ‘other’ patients with CP and (b) Indigenous index cases and matched controls. Statistical significance was set at p≤0.05.

## Results

The index case cohort consisted of 2503 adults with at least one SA public hospital or ED visit with a primary or secondary diagnosis of CP in adulthood during the 20-year period between 1 June 2000 to 30 June 2019. Control groups consisted of 3277 individuals in the type 1 diabetes control group, 5006 members of the type 2 diabetes control group, 7509 members of the general population and 12 011 individuals with a diagnosis of AP in the study time frame (table 1). Planned control numbers in the type 1 diabetes and type 2 diabetes were unable to be achieved given the stringent age- and sex-matching process and a larger-than-expected cohort of individuals with CP. The case group had a significantly lower median IRSAD score than all of the control groups except the type 2 diabetes control group (table 1).

**Table 1:** Characteristics of index cohort and matched controls: demographic details of CP index cases and control populations identified by ICD-10 code within South Australia between 1 June 2000 and 30 June 2019.

Variables	CP	General public	Type 1 diabetes mellitus	Type 2 diabetes mellitus	AP
Total	2503	7.509	3277	5006	12 011
Male	1564 (62.5%)	4692 (62.5%)	2048 (62.5%)	3129 (62.5%)	5905 (49.2%)
Female	939 (37.5%)	2817 (37.5%)	1229 (37.5%)	1877 (37.5%)	6106 (50.8%)
Self-identified as Aboriginal or Torres Strait Islander	358 (14.3%)	90 (1.2%)	85 (2.6%)	590 (11.8%)	745 (6.2%)
IRSAD Centile median (IQR)	3 (1–6)	5 (2–8)*	4 (2–7)*	3 (1–6)	4 (2–7)*

* P<0.0001 versus CP cases.

APacute pancreatitisCPchronic pancreatitisICD-10International Classification of Diseases 10th RevisionIRSADIndex of Relative Socio-economic Advantage and Disadvantage

A summary of the index case cohort is displayed in table 2. The median follow-up from initial diagnosis of CP was 9 years (IQR: 4 to 15 years). CP was more common in males than females (p<0.001) and tended to be diagnosed in mid-life (median 53 years, IQR=41 to 66 years). Around 15% of individuals self-identified as Aboriginal or Torres Strait Islander people. Most cases were identified as ICD-10 code K86.1, or ‘other’ CP. A range of key health indicators stratified by case-control status are shown in table 3. AP was equally common in males and females. Patients with CP had significantly more hospital and ED visits, days in hospital, procedures, ICU admissions and hours spent in ICU compared with patients with AP and the general population (p<0.001). Within the case cohort, 92 (3.6%) patients were diagnosed with pancreatic cancer over the 20-year period, with 6 (6.5%) of these identifying as Aboriginal and/or Torres Strait Islander people. This was compared with a total of 71 (0.44%) of type 1 diabetes, type 2 diabetes and general population controls (an overall cohort of 15 972 individuals) receiving a diagnosis of pancreatic cancer within the same period. Comorbid CP was thus associated with approximately eightfold increased risk of pancreatic cancer relative to the control groups (crude OR=8.27, 95% CI 6.05 to 11.30, p<0.0001). Within the AP cohort, 302 (2.5%) individuals received a diagnosis of pancreatic cancer within the study period.

**Table 2:** Characteristics of index cohort: detailed characteristics of the CP cohort identified by data linkage in South Australia between 2000 and 2019.

Median age of diagnosis (years)	53 (41–66)
Previous diagnosis of AP	1772 (70.1%)
‘Alcohol-induced’ CP	940 (37.5%)
Male	693 (73.7%)
Female	247 (26.2%)
Indigenous	185 (51.7%)
‘Other’ CP	1563 (62.4%)
Male	871 (55.7%)
Female	692 (44.3)
Indigenous	173 (48.3%)
Median time in dataset (years)	9 (4–15)
Coded deaths in hospital	468 (18.7%)
Diagnosis of pancreatic cancer (2000 – 2019)	92 (3.6%)

APacute pancreatitisCPchronic pancreatitis

**Table 3:** Case-control group comparisons for key health indicators

Group	n	Hospital visits* median (IQR)	ED visits* median (IQR)	Hospital procedures† median (IQR)	Hospital days‡ median (IQR)	At least 1 ICU admission§ n (%)	Hours in ICU ¶ median (IQR)
CP	2503	11 (5–21.75)	8 (3–19)	46 (19–99)	18 (7–36)	984 (39.3%]	83 (33–215)
General population controls	7509	1** (0–4)	1** (0–4)	6** (2–27)	5** (2–13)	746 (9.9%)**	61** (25–147)
Type 1 diabetes mellitus	3277	2** (1–4)	1** (0–4)	6** (2–15)	3** (1–7)	369 (11.3%)**	46** (21–123)
Type 2 diabetes mellitus	5006	5** (2–11)	4** (1–9)	15** (5–50)	10** (4–21)	1377 (27.5%)**	65** (26–149.5)
AP	12 011	6** (3–12)	5** (2–11)	19** (7–52)	11** (4–23)	2943 (24.7%)**	70** (30–182)

* Total number of visits over 20-year study period.

† Total number of in-hospital procedures over 20-year study period.

‡ Total number of days spent in hospital over 20-year study period.

§ ≥1 visits to an ICU over 20-year study period.

¶ Total number of hours spent in an ICU over 20-year study period.

** P<0.001 compared with CP group.

APacute pancreatitisCPchronic pancreatitisEDemergency departmentICUintensive care unit

Patients with CP coded as having alcohol-induced CP had a younger median age of diagnosis (median 47 years, IQR=39 to 55) compared with those coded as ‘other’ CP (median 58 years, IQR=44 to 73) (p<0.001). The majority (73.7%) of the alcohol-induced patients with CP were male in contrast to the ‘other’ CP group (55.7%) (p<0.001). Other than median hours in ICU, the alcohol-induced CP group had consistently higher healthcare-associated burden than the ‘other’ CP group (all p<0.001; table 4). Indigenous patients with CP had a younger median age of diagnosis at 41 years (IQR=33 to 50) than the rest of the CP cohort (p<0.001). Indigenous patients with CP had more ED visits, procedures and hours in ICU than both non-Indigenous individuals with CP and Indigenous individuals in the control groups (all p<0.001; table 5). Indigenous patients with CP were also more likely to be diagnosed with ‘alcohol-induced’ CP than non-Indigenous patients (p<0.001). Indigenous patients with CP had a significantly lower median IRSAD than non-Indigenous individuals with CP but did not differ from Indigenous individuals in the control groups (Table 5). Indigenous patients with ‘alcohol-induced’ [K86.0] CP (median IRSAD=2 (IQR=1 to 5)) did not significantly differ in median IRSAD from ‘other’ CP (K86.1) (median IRSAD=1 (IQR=1 to 3); p=0.24).

**Table 4:** Alcohol-induced patients with CP compared with ‘other’ patients with CP

Group	n	Hospital visits* median (IQR)	ED visits* median (IQR)	Hospital procedures† median (IQR)	Hospital days‡ median (IQR)	At least 1 ICU admission§ n (%)	Hours in ICU¶ median (IQR)
Alcohol-induced CP	940	13 (6–27)	11 (4–27)	55 (26–118)	21 (8.8–38)	420 (44.7%)	85 (39–220)
Other CP	1563	9** (5–19)	7** (2–15)	40** (17–90)	17** (7–34)	545** (34.9%)	80 (28–211)

* Total number of visits over 20-year study period.

† Total number of in-hospital procedures over 20-year study period.

‡ Total number of days spent in hospital over 20-year study period.

§ ≥1 visits to an ICU over 20-year study period.

¶ Total number of hours spent in an ICU over 20-year study period.

** P<0.001 compared with alcohol-induced group with CP.

CPchronic pancreatitisEDemergency departmentICUintensive care unit

**Table 5:** Indigenous case-control comparators for key health indicators

Group	N	Hospital visits* median (IQR)	ED visits* median (IQR)	Hospital procedures† median (IQR)	Hospital days‡ (IQR)	At least 1 ICU admission§ N (%)	Hours in ICU¶^,^ median (IQR)	IRSAD centile median (IQR)
Indigenous CP	358	16 (7–32)	13 (3–29)	19 (7–36)	53 (24–125)	165(41%)	98 (43–234)	2 (1-4)
Non-Indigenous CP	2145	10** (5–20)	7** (3–17)	18** (7–36)	44 (18–95)	838 (38%)	82** (30-214)	4 (2-8)**
Indigenous general population	90	6** (3–14)	5** (3–14)	9.5** (3–20)	14.5** (5–56)	24** (27%)	53.5 (23-137)	2 (1-5)
Indigenous type 1 diabetes mellitus	85	1** (1–3)	0** (0–3)	3** (0–7)	6** (2–13)	14** (16%)	32.5 (22-135)	2 (1-5)
Indigenous type 2 diabetes mellitus	590	6** (2–16)	4** (1–13)	11** (5–26)	20** (8–66)	199 (34%)	71(34-156)	2 (1–5.5)
Indigenous AP	745	10** (4–23)	10** (3–23)	14** (6–26)	29** (12–75)	262 (35%)	92(39-215)	2 (1-5)

* Total number of visits over 20-year study period.

† Total number of in-hospital procedures over 20-year study period.

‡ Total number of days spent in hospital over 20-year study period.

§ ≥1 visits to an ICU over 20-year study period.

¶ Total number of hours spent in an ICU over 20-year study period.

** P<0.001 compared with CP group.

APacute pancreatitisCPchronic pancreatitisEDemergency departmentICUintensive care unit

SA Census data indicated an adult population of 1 282 968 in SA in 2016.^19^ Accounting for patients who died in hospital, the crude prevalence estimate for the CP cohort in 2019 in the adult population of SA was 195.1 per 100 000 persons. Age-adjusted rate was 189.1 per 100 000 persons. There was an average of 133.7 first presentations with CP per annum in SA, giving a crude incidence estimate of 10.4 per 100 000 adult persons per year. This does not account for patients who were diagnosed with CP prior to the beginning of the study period (1 June 2000). CP incidence was stable over the study period.

## Discussion

This is the first whole-of-population study to describe the epidemiology and healthcare burden of adult CP in Australia. We have investigated the characteristics of adult-onset CP and compared those characteristics with age- and sex-matched controls from the general population and with age- and sex-matched controls with either type 1 diabetes or type 2 diabetes. These findings suggest that CP in adulthood is a remarkably burdensome condition which disproportionally affects Indigenous people.

The estimated crude prevalence of CP does not account for deaths outside of hospital, changes in population size and patients moving out of SA, meaning the true prevalence is likely lower than reported. An international, multicentre study of 2015 patients with CP demonstrated a 10-year survival rate of 70%, and a 20-year survival of 45% after diagnosis.^26^ A higher death rate can therefore be assumed than demonstrated by in-hospital data alone. Extrapolating a death rate of either equal or double in-hospital deaths indicates an estimated prevalence in the study cohort of approximately 100 per 100 000. These results are consistent with international data available regarding the epidemiology of CP. Estimates of the annual incidence of CP range between 5 and 14 per 100 000 per year,^27^ consistent with our result of 10.4 per 100 000. Prevalence estimates in the literature range from 50 per 100 000^2^ to 143 per 100 000.^28^ Our higher prevalence estimates may be explained by the impact of the disproportionate prevalence in the Indigenous Australian population, given previous epidemiological analyses have been performed in primarily European-ancestry populations. Consistent with our findings, most studies have demonstrated that males are more affected than females, with a mean age of diagnosis in the fifth to sixth decade.^29^ Our data demonstrated that AP diagnoses were equivalent between males and females, suggesting males may be at higher risk of progressing to CP.

Alcohol-induced CP has generally been implicated in previous epidemiological studies as being a causal factor in 70–80% of adult CP cases.^30^,^35^ However, these estimates depend on local diagnostic protocols where reporting bias may be present and should thus be interpreted cautiously.^36^ In contrast, our study found that this diagnosis comprised the minority (37.5%) of adult CP cases. This suggests that aetiological factors other than alcohol may be more important in the Australian population.

The Indigenous population was significantly over-represented within the CP cohort. As of the 2016 SA Census estimate, 1.5% of the adult population identified as Aboriginal or Torres Strait Islander (n=19 207).^19^ This contrasts with 14.3% in the study dataset who identified as Indigenous (n=358). The Indigenous population with CP was additionally found to have increased healthcare requirements. There are broader socioeconomic and modifiable risk factors to consider that may contribute to this disparity,^37^ as suggested by the lower IRSAD in the Indigenous CP cases. However, the median IRSAD in ‘other’ CP (K86.1) cases was lower than the median IRSAD in ‘alcohol-related’ CP (K86.0) cases, suggesting that there was not a strong confounding effect of SES on alcohol-induced CP in Indigenous CP cases. The younger median age of diagnosis may also suggest a higher rate of inherited factors contributing to pancreatitis in this population group. This is not limited to Indigenous Australians; a 2018 systematic review found that pancreatic diseases are more common in Indigenous populations globally.^38^ We have recently described this association in SA children, with 24 out of 73 (32.8%) children with CP identifying as Aboriginal and/or Torres Strait Islander.^39^ Most paediatric CP results from inherited genetic factors, consistent with a strong biological basis for the elevated prevalence of CP in the Indigenous population.^40^ There is clearly a need for more detailed investigations of CP in the Indigenous Australian population.

The role of CP as a risk factor for pancreatic cancer has been less extensively researched compared with more well-documented risk factors such as tobacco smoking, alcohol intake and diabetes mellitus^41^; however a recent systemic review and meta-analysis found that those with a diagnosis of CP have a 16-fold higher risk of pancreatic cancer within 2 years of initial diagnosis when compared with those without CP.^42^ The degree of risk increased over time to threefold at 9 years of follow-up, but remained significantly elevated.^42^ We found that pancreatic cancer incidence was significantly increased among patients with CP. The incidence of pancreatic cancer (3.6%) among patients with CP extrapolates to a pancreatic cancer incidence of 180 per 100 000 individuals with CP per year, far higher than the incidence of 6.4 per 100 000 per year in the general Australian population.^43^ This is consistent with international studies demonstrating that 2–4% of patients with CP will develop pancreatic cancer.^44 45^

The hospital presentations identified within the dataset represented how much each individual within each patient cohort accessed healthcare, rather than disease-specific presentations. Our study has shown that adult-onset CP carries a high healthcare burden in SA. Key indicators such as 11-fold more hospital visits, 8-fold increase in diagnoses of pancreatic cancer, 8-fold more ED visits and almost 8-fold more total days spent in hospital than the general population demonstrate the significant impact of this condition.

### Strengths and limitations

A relative strength of the study was the ability to capture whole-of-population CP diagnoses in SA public hospitals. Reliable population-wide data regarding CP disease burden are scarce.^36^ Previous epidemiological studies^2 4 46 47^ have tended to extrapolate from smaller cross-sectional community data to produce state or national estimates. Our study has several limitations inherent in the use of linkage to government administrative data including reliance on appropriate ICD-10 and other coding on hospital discharge. ICD-10 coding is limited in CP and does not offer any further stratification than ‘alcohol-induced’ or ‘other’ CP. Information bias may arise from human administrative error, which in combination with the difficulty of diagnosing CP versus recurrent AP may lead to either over- or underestimations of prevalence and incidence. In July 2020, SA-NT DataLink conducted an empirical review of the linkage quality within the Master Linkage File for the SA data sources. This published report determined a 99.6% accuracy (0.4% false positive rate) with an upper bound of the 95% CI at 0.9%, while the false negative rate or ‘missed link’ rate was 0.9% (or 99.2% accurate) within an upper bound on the error rate at 1.7%.^48^ Our datasets comprised only public hospital data, with private hospitals not yet providing information to SA-NT DataLink. Additionally, a patient with CP must have visited a SA public hospital to be represented in the dataset as a ‘case’ meaning that there may be a cohort of patients with CP who have been missed in the prevalence estimate, having solely being managed in the outpatient or primary care setting. Consequently, the dataset may overestimate burden in that the more unwell patients are more likely to be managed in hospital. Mitigating this is the difficulty of reaching a CP diagnosis. This typically results from hospitalisation for significant abdominal pain, requiring imaging and exclusion of other diagnoses. The major strength of this study was its high statistical power to find modest effect sizes related to the volume of unbiased data, with over 2500 index cases and large control group comparators.

## Conclusions

Our data have allowed for estimations of the prevalence and incidence of a condition previously undescribed epidemiologically within Australia. It is evident that CP is burdensome for the SA healthcare system, with significantly more visits to hospital, and higher hospitalisation-related events in the CP case cohort than any control group. Indigenous adults are disproportionately affected in SA. There are limited therapeutic interventions available once CP has developed, and as such early detection and intervention in the primary care setting is crucial in reducing the demonstrable burden of this disease.

## Data Availability

No data are available.
