# Supplementary material for: Epidemiology and burden of adult chronic pancreatitis in South Australia: a 20-year data linkage study
Source: BMJ Open. 2025 Mar 6;15(3):e089297. doi: 10.1136/bmjopen-2024-089297 (PMC11887304; doi:10.1136/bmjopen-2024-089297)
Supplement: Supplementary file 1 [file bmjopen-2024-089297.reviewer_comments.pdf]

PEER REVIEW HISTORY

BMJ Open publishes all reviews undertaken for accepted manuscripts. Reviewers are asked to complete a checklist review form and are provided with free text boxes to elaborate on their assessment. These free text comments are reproduced below.

ARTICLE DETAILS

Title (Provisional)

The epidemiology and burden of adult chronic pancreatitis in South Australia: a 20 year data linkage study

Authors

Bampton, Tristan J.; Chen, John W.; Brown, Alex; Barnett, Meghan I.; Coates, P. Toby; Palmer, Lyle John

---

VERSION 1 - REVIEW

**Reviewer**	**1**
**Name**	**Mole, Damian**
**Affiliation**	**University of Edinburgh MRC Centre for Inflammation Research**
**Date**	**22-Jun-2024**
**COI**	**I have no competing interests with this paper to declare**

Despite this reviewers constructive comments, the authors have decided not to conduct the analysis of confounding of social deprivation category in indigenous peoples with chronic pancreatitis. Their excuse is that it would delay the publication of their results, but they have take 15 months to do extremely limited minor revisions and have done no further analysis, so this reason as provided is not robust, unless there are other significant extenuating circumstances which have not been communicated. It is not a satisfactory response. If the authors were so desperate to publish this paper, it should not have taken over a year to resubmit an essentially unchanged manuscript.

They have made no effort to analyse confounding of increased problem alcohol use potentially associated with indigenous peoples, despite this reviewer's clear recommendation.

Lastly, inserting a new claim of "Most paediatric CP results from inherited genetic factors, consistent with a strong biological basis for the elevated prevalence of CP in the Indigenous population.38", without a Mendelian Randomisation analysis is weak, especially from a group with strengths in genetic epidemiology.

VERSION 1 - AUTHOR RESPONSE

Reviewer: 1

Comments to the Author:

Despite this reviewers constructive comments, the authors have decided not to conduct the analysis of confounding of social deprivation category in indigenous peoples with chronic pancreatitis. Their excuse is that it would delay the publication of their results, but they have take 15 months to do extremely limited minor revisions and have done no further analysis, so this reason as provided is not robust, unless there are other significant extenuating circumstances which have not been communicated. It is not a satisfactory response. If the authors were so desperate to publish this paper, it should not have taken over a year to resubmit an essentially unchanged manuscript.

They have made no effort to analyse confounding of increased problem alcohol use potentially associated with indigenous peoples, despite this reviewer's clear recommendation. Lastly, inserting a new claim of "Most paediatric CP results from inherited genetic factors, consistent with a strong biological basis for the elevated prevalence of CP in the Indigenous population.38", without a Mendelian Randomisation analysis is weak, especially from a group with strengths in genetic epidemiology.

Response: We apologise to the reviewer for failing to fully respond to his comments in our initial revision. In response to the reviewer's concerns, we have obtained a measure of SES in each of our study participants - the Index of Relative Socio-economic Advantage and Disadvantage (IRSAD). The IRSAD is produced by the Australian Bureau of Statistics and ranks areas in Australia according to relative socio-economic advantage and disadvantage based on information from the 2016 Australian Census. We have updated our methods and results sections to include this measure. The results showed that median IRSAD was significantly lower in the CP cases than all of the control groups with the exception of the type 2 diabetes controls. This difference was largely driven by the Indigenous CP cases, whose median IRSAD was significantly lower than that of non-Indigenous CP cases. However, the median IRSAD in 'other' CP (K86.1) cases was lower than the median IRSAD in 'alcohol related' CP (K86.0) cases suggesting that there was not a strong confounding effect of SES on alcohol induced CP in Indigenous CP cases. We have modified our discussion of the possible causes of the higher prevalence of CP in the Indigenous population in light of our own analyses and the reviewer's comments.
